# Elevation of small, dense low density lipoprotein cholesterol—a possible antecedent of atherogenic lipoprotein phenotype in type 2 diabetes patients in Jos, North-Central Nigeria

**DOI:** 10.1186/s12907-017-0065-9

**Published:** 2017-12-06

**Authors:** Kenneth O. Inaku, Obasola O. Ogunkeye, Fayeofori M. Abbiyesuku, Evelyn K. Chuhwak, Christian O. Isichei, Lucius C. Imoh, Noel O. Amadu, Alexander O. Abu

**Affiliations:** 1 Department of Chemical Pathology, Faculty of Medicine, College of Medical Sciences, University of Calabar, Calabar, Cross River State Nigeria; 2 Department of Chemical Pathology, Faculty of Medical Sciences, University of Jos, Jos, Plateau State Nigeria; 3 Department of Chemical Pathology, Faculty of Medicine, University of Ibadan, Ibadan, Oyo State Nigeria; 4 Department of Internal Medicine, Faculty of Medical Sciences, University of Jos, Jos, Plateau State Nigeria; 5 Department of Chemical Pathology, University of Jos Teaching Hospital, Jos, Plateau State Nigeria

**Keywords:** Type 2 diabetes mellitus, Dyslipidaemia, Small, dense LDL-C, Atherogenesis, High density lipoprotein

## Abstract

**Background:**

The global prevalence of type 2 diabetes is increasing. Dyslipidaemia is a known complication of diabetes mellitus manifesting frequently as cardiovascular diseases and stoke. Elevation of small, dense low density lipoprotein has been recognised as a component of the atherogenic lipoprotein phenotype associated with cardiovascular complications. We speculate that the elevation of this lipoprotein particle may be the antecedent of the atherogenic lipoprotein phenotype. This study therefore aims to determine the pattern of dyslipidaemia among diabetes mellitus patients in Jos, North-Central Nigeria.

**Methods:**

One hundred and seventy-six patients with type 2 diabetes and 154 age-matched controls were studied. The patients with diabetes were regular clinic attenders and had stable glycaemic control. None were on lipid-lowering therapy. Anthropometric indices, blood pressure, and lipids (including total cholesterol, high density lipoprotein cholesterol, and triglyceride) were measured by chemical methods using the Hitachi 902 analyzer. Low density lipoprotein cholesterol was calculated using the Friedewald’s equation. Small, dense low density lipoprotein cholesterol, −sdLDL-C was measured using the precipitation method by Hirano et al. Means of the different groups were compared using EPI Info and a *P*-value of <0.05 was accepted as significant difference.

**Results:**

Total cholesterol, low density lipoprotein cholesterol, triglyceride and small, dense lipoprotein cholesterol were all significantly higher in diabetes patients than controls except high density lipoprotein cholesterol. The percentage of LDL-C as sdLDL-C among the diabetes versus control group was 45% ± 17.79 v 32.0% ± 15.93. Serum sdLDL-C concentration was determined to be 1.45 ± 0.64 among diabetes patients and 0.8 ± 0.54 among control subjects. 75% of diabetes patients had hypertension and were taking blood pressure lowering medications.

**Conclusion:**

The classical atherogenic lipoprotein phenotype was not demonstrated among subjects with type 2 diabetes mellitus in this study, but the elevation of serum small dense low density lipoprotein cholesterol in patients with sustained hypertension suggests the establishment of atherogenic complications among our diabetes patients.

## Background

Type 2 diabetes is an increasingly common chronic illness with an accompanying risk of cardiovascular complications [1]. In 2015, an estimated 415 million people worldwide were said to be living with diabetes representing 8.8% of the global adult population. By 2040, that number is expected to rise to 642 million [2]. With some 75% of people with diabetes living in low and middle income countries, diabetes is no longer a disease of the Western countries. Nigeria has a national prevalence of about 1.9% [2] although this may fall below the true prevalence of the disease in Nigeria due to the poor health care services especially in rural areas where majority of ailments go undiagnosed.

Diabetes mellitus is frequently associated with serious health complications and premature deaths. Globally, it is estimated to account for 50 million deaths annually at a frequency of 1 death every 6 s [2]. Approximately 80% of all diabetes associated mortality and hospitalisations can be attributed to cardiovascular complications [3]. Dyslipidaemia in type 2 diabetes may exist alone or in association with metabolic syndrome, and this association increases cardiovascular risk [4]. The typical pattern of diabetic dyslipidaemia consists of elevated very low density lipoprotein (VLDL) triglycerides, low high density lipoprotein cholesterol (HDL-C) and a predominance of small, dense low density lipoprotein cholesterol (sdLDL-C) [5]. This diabetic dyslipidaemia was first described in 1990 by Austin et al. as a risk conferring lipoprotein profile termed “atherogenic dyslipidaemia” or “the atherogenic lipoprotein phenotype” (ALP) [6]. The increase in the number of atherogenic particles reflected by high apolipoprotein B (apoB) concentrations may contribute to an increased cardiovascular disease (CVD) mortality in people with diabetes (phenotype B) [4, 7]. This is in contrast to phenotype A pattern, in which large, more buoyant LDL predominates.

LDL-C remains the target for diagnosing and monitoring dyslipidaemia in type 2 diabetes [8]. The bulk of research on dyslipidaemias in Nigeria, both in the general population and specifically among type 2 diabetes patients, has concentrated on measuring traditional lipid indices such as total cholesterol (TC), LDL-C, HDL-C and triglyceride (TG) [9]. We are unaware of studies in Nigeria that have estimated LDL-C sub-fractions in diabetes versus controls. Since lipids can be affected by diet, environment and lifestyle, there is a need to investigate the metabolism of sdLDL-C as a component of dyslipidaemia among people with diabetes living in Jos, Nigeria. We hypothesise that the level of sdLDL-C will be higher in diabetes subjects than controls.

## Methods

### Study area

The protocol for this study was approved by the Ethics Committee of the Jos University Teaching Hospital (JUTH). The study area was Jos and Bukuru metropolis in the Plateau State of Nigeria. The population is made up of civil servants, students and traders.

### Study design and population

This is a descriptive, cross-sectional study. Subjects aged 35-65 years were recruited from type 2 diabetes patients attending both the Medical outpatient department (MOPD) and the General Outpatient department (GOPD) of the Jos University teaching hospital (JUTH) from June to September 2012. They were selected by the attending physician after being confirmed to be regular attendees in clinics for at least 3 consecutive visits, taking prescribed medications and never having been on lipid lowering medications. No patient was enrolled twice. Controls were individuals without diabetes or hypertension drawn from hospital staff, patients’ relatives and members of the general public who had no symptoms suggestive of diabetes mellitus or hypertension. They were examined to confirm their status. All participants gave written informed consent before enrolling in the study.

Each participant was administered a questionnaire. The questionnaire was completed by the individuals if they could read and write English language and otherwise by the researcher or his trained assistant. Demographic and anthropometric indices such as age, sex, weight and height, blood pressure were recorded. Blood pressure was recorded while patient was sitting using a table mercury column sphygmomanometer after about 5-10 min of rest. Hypertension was defined by systolic or diastolic blood pressure above 139/89 mmHg on more than one occasion or taking blood pressure lowering medications. Body mass index was calculated from the formula: weight (kg)/height (m^2^). A history of past or present use of lipid-lowering medications was also noted.

### Exclusion criteria

1) individuals on known lipid-lowering drugs; **2)** subjects with a triglyceride level above 4.5 mmol/L; **3)** pregnant women; 4) individuals with acute or chronic illness (except type 2 diabetes and hypertension); 5) anyone who did not give written informed consent; 6) any subject on admission to hospital irrespective of type or nature of illness. 6) Hypertensive controls without diabetes.

## Sample collection and analysis

### Blood sampling

About 5 ml of venous blood was collected into a plain bottle from each subject following an overnight fast of at least 10 h. The blood was allowed to clot and retract at room temperature before centrifuging at 3500 rpm for 5 min. Serum was separated and stored at -20 °C and used for the assays within 7 days of sampling. Refrigerator temperature recording was done twice daily to ensure adequate preservation of the samples. This is a part of the daily routine in the AIDS Prevention in Nigeria (APIN) Laboratory. Blood samples were analysed for total cholesterol (TC), high density lipoprotein cholesterol (HDL-C), and triglyceride (TG) by automated colorimetric enzymatic analysis using Cobas (Roche Diagnostics GmbH, Sandhofer Strasse 116, Mannheim, Germany) commercial kits on the Roche/Hitachi 902 automatic analyser (Hitachi High-Technology Corporation, Minato-ku, Tokyo 105-8717, Japan). Low density lipoprotein cholesterol was calculated using Friedewald’s formula [10] provided the TG level was <4.5 mmol/L. Small, dense low density lipoprotein cholesterol (sdLDL-C) was isolated using the precipitation method of Hirano et al. [11]. The precipitation reagent (0.1 ml) contained 150 U/ml of heparin-sodium salt (Sigma) and 90 mmol/l of MgCl_2_ (Sigma), and was added to 0.1 ml of plasma, mixed, and incubated for 10 min at 37 °C. The samples were then placed in an ice bath for 15 min, and then the precipitate was collected by centrifugation at 15000 rpm for 15 min at 4 °C. Aliquots of the supernatant were then used to measure the cholesterol concentration by the same kit method used for total cholesterol. The within- and between-run coefficient of variations (CVs) for cholesterol measurements was 0.48% and 4.72% and for TG measurements it was 0.45% and 9.9% respectively. We calculated large buoyant LDL-C as LDL-C – sdLDL-C, and we also calculated the percentage of LDL-C as sdLDL-C as sdLDL-C/LDL-C × 100.

## Data analysis

Data was entered into Microsoft Excel and imported into Epi Info™ version 7 [12] for analysis. Socio-demographic characteristics of the diabetes and control groups were analyzed for differences, using unpaired Student’s t-tests. Mean (SD) or 95% confidence interval (CI) of the mean was computed for all lipid variables. A comparison of means across groups where there are more than 2 groups as in Table 7 was made using analysis of variance (ANOVA). *P*-values <0.05 were considered to be statistically significant for all tests.

## Results

The study cohort consisted of 176 patients with type 2 diabetes and 154 controls aged 35-65 years. Among the patients with diabetes, there were 60 men (34%) and 116 women (65%), while in the controls there were 69 men (45%) and 85 women (55%). The duration of diabetes ranged from 1 to 22 years, with a mean of 6.48 ± 4.8 years. Among these, 141 (80%) have had diabetes for at least 10 years while 35 (20%) have been diagnosed over 10 years prior to the time of this study. The age and weight between the diabetes and control groups were not statistically significantly different since they were age matched. However, the diabetes group had a statistically significantly higher body mass index (BMI), systolic and diastolic blood pressure than the controls (Table 1). One hundred and thirty-two (75%) of the patients with diabetes had hypertension and were being treated with blood pressure lowering medications. The duration of hypertension ranged from 1 to 31 years with an average of 7.40 ± 7.81. Nine (0.05%) of the diabetes subjects reported a past history of cardiovascular event ranging from transient ischemic attack (TIA) to stroke. There was no report of a history of myocardial infarction among the subjects. Diabetes was being managed with metformin as first line with sulphonylureas or thiazolidiandiones such as chlopropamide and pioglitazone respectively as adjunct. Five (0.03%) patients were treated with insulin in combination with metformin and 2 (0.01%) patients were being managed with insulin only. Two patients did not need medications to control their blood glucose level and were on diet modification only. None of the diabetes subjects was taking lipid lowering medications at least 6 month prior to, and during the study period.

**Table 1:** Demographics and some indices of diabetes patients and controls

	Diabetics	Controls	*P*-value
Characteristics	(*n* = 176)	(*n* = 154)	
Age (years)	55 ± 8.5	54 ± 7.3	0.89
Weight (Kg)	74 ± 14.9	72 ± 11.7	0.14
BMI (Kg/m^2^)	28.5 ± 5.0	26.7 ± 4.6	< 0.01
Systolic BP (mmHg)	138 ± 29.0	121 ± 17.9	< 0.01
Diastolic BP (mmHg)	87 ± 15.8	80 ± 10.3	< 0.01

Values are expressed as mean ± SD. Unpaired Student’s t-tests, *P* < 0.05 = significant

We compared lipid parameters between patients with diabetes and controls (Table 2). Mean serum concentrations of TC (5.16 ± 1.31 v 4.35 ± 1.02 mmol/l, *P* < 0.01), TG (1.42 ± 0.63 v 1.00 ± 0.56 mmol/L, *P* < 0.01), LDL-C (3.31 ± 1.20 v 2.60 ± 0.91 mmol/L, *P* < 0.01) and small, dense LDL-C (1.45 ± 0.64 v 0.80 ± 0.54 mmol/l, *P* < 0.01) were all higher in patients with diabetes than control. Only HDL-C (1.21 ± 0.36 v 1.26 ± 0.40 mmol/l, *P* = 0.19) and large buoyant LDL-C (1.86 ± 1.09 v1.84 ± 0.93 mmol/l, *P* = 0.85) were not significantly different. The percentage of LDL-C as sdLDL-C among the diabetes patients and controls was 45% and 32%, respectively. All the different lipid and lipoprotein ratios examined were higher among the diabetes patients than the controls except HDL-C/LDL-C, which was higher among controls (0.56 ± 0.36) than the diabetes patients (0.41 ± 0.9 mmol/l).

**Table 2:** Serum lipid concentration in diabetes patients and controls

	Diabetics	Controls	*P*-value
Variable	(*n* = 176)	(*n* = 154)	
	Mean ± SD	Mean ± SD	
Total Cholesterol (mmol/L)	5.16 ± 1.31	4.35 ± 1.02	< 0.01
Triglycerides (mmol/L)	1.42 ± 0.63	1.00 ± 0.56	< 0.01
HDL-C (mmol/L)	1.21 ± 0.36	1.26 ± 0.40	0.19
LDL-C (mmol/L)	3.31 ± 1.20	2.60 ± 0.91	< 0.01
sdLDL-C (mmol/L)	1.45 ± 0.64	0.80 ± 0.54	< 0.01
lbLDL-C (mmol/L)	1.86 ± 1.09	1.84 ± 0.93	0.85
% LDL-C as sdLDL-C	45.0 ± 17.79	32.0 ± 15.93	< 0.01
TC/HDL-C	4.63 ± 2.40	3.69 ± 1.19	< 0.01
HDL-C/LDL/C	0.41 ± 0.19	0.56 ± 0.36	< 0.01
LDL-C/HDL-C	3.05 ± 2.22	2.29 ± 1.04	< 0.01

Values are expressed as mean ± SD, Unpaired Student’s t-tests, *P* < 0.05 = significant

Table 3 compares the mean lipid parameters in the diabetes group between men and women. The total cholesterol, HDL-C, LDL-C and sdLDL-C and lbLDL-C were all statistically significantly higher in women than men with diabetes. The TG value is the same in women as in men with diabetes. Men with diabetes are more likely to have up to 47% (95% CI 42.84–52.16) of their LDL-C as small dense LDL-C. The difference in the percentage of LDL-C as sdLDL-C in women and men (44.83% v 47.50%, *P* = 0.37) did not reach statistical significance.

**Table 3:** Serum lipid concentration in diabetic men and women

Variable	Men	Women	*P*–value
	(*n* = 60)	(*n* = 116)	
Total Cholesterol (mmol/l)	4.75 (4.45–5.05)	5.37 (5.13–5.16)	< 0.01
Triglycerides (mmol/l)	1.39 (1.23–1.54)	1.43 (1.32–1.55)	0.68
HDL–C (mmol/l)	1.11 (1.04–1.19)	1.26 (0.99–1.33)	0.01
LDL–C (mmol/l)	3.02 (2.76–3.28)	3.46 (3.23–3.69)	0.01
sdLDL–C (mmol/l)	1.38 (1.23–1.53)	1.48 (1.36–1.60)	0.32
lbLDL–C (mmol/l)	1.64 (1.41–1.87)	1.98 (1.77–2.19)	0.03
%LDL as sdLDL	47.50 (42.84–52.16)	44.83 (41.39–48.27)	0.37

Values are expressed as mean (95% Confidence interval of the mean); Unpaired Student’s t-tests, *P* < 0.05 = significant

Table 4 compares serum lipid concentrations between male and female controls. The mean total cholesterol among men was 4.38 mmol/l (95% CI 4.12–4.63), and 4.32 mmol/l (95% CI 4.11–4.53) in women. The difference was not statistically significant (*P* < 0.75). Triglyceride were higher in men (1.11 mmol/l, 95% CI 0.97–1.25) than in women (0.92 mmol/l, 95% CI 0.81–1.02) and the difference was statistically significant (*P* = 0.03). The mean HDL-C was not different in women (1.32 mmol/l, 95% CI 1.22–1.42) than men (1.20 mmol/l, 95% CI 1.12–1.27, *P* = 0.05). LDL-C was the same (*P* = 0.51) in men (2.7 mmol/l, 95% CI 2.50–2.90) as in women (2.60 mmol/l, 95% CI 2.40–2.80). This was also the case with sdLDL-C, which was the same (*P* = 0.39) in women (0.83 mmol/l, 95% CI 0.70-0.96) as in men (0.76 mmol/l, 95% CI 0.56-0.86). The percentage of LDL-C as sdLDL in women was the same (*P* = 0.56) at 33.89% (95%CI 28.86–38.93) as in men at 31.46% (95% CI 24.97–37.96).

**Table 4:** Serum lipid concentrations in men and women controls

Variable	Men	Women	*P*-value
	(*n* = 69)	(*n* = 85)	
Total cholesterol (mmol/l)	4.38 (4.12–4.63)	4.32 (4.11–4.53)	0.75
Triglycerides (mmol/l)	1.11 (0.97–1.25)	0.92 (0.81–1.02)	0.03
HDL–C (mmol/l)	1.20 (1.12–1.27	1.32 (1.22–1.42)	0.05
LDL–C (mmol/l)	2.70 (2.50–2.90)	2.60 (2.40–2.80)	0.51
sdLDL–C (mmol/l)	0.76 (0.65–0.86)	0.83 (0.70–0.96)	0.39
lbLDL–C (mmol/l)	1.94 (1.72–2.16)	1.77 (1.57–1.96)	0.25
%LDL as sdLDL	31.46 (24.97–37.96)	33.89 (28.86–38.93)	0.56

Values are expressed as mean (95% confidence interval), Unpaired student’s t-test, *P* < 0.05 = significant

The serum lipid concentrations were compared among men with diabetes and men without diabetes (Table 5). TC, HDL-C, LDL-C, and lbLDL-C were not statistically significantly different between the two groups. TG, sdLDL-C and percentage of LDL-C as sdLDL reached statistical significant difference between the groups. Men with diabetes are likely to have as much as 47.50% (95% CI 42.84%–52.16%) of LDL existing as sdLDL-C.

**Table 5:** Serum lipid concentrations in men with diabetes compared with men without diabetes

Variable	Diabetes	Controls	*P*-value
	(*n* = 60)	(*n* = 69)	
Total cholesterol (mmol/l)	4.75 (4.45-5.05)	4.38 (4.12-4.63)	0.068
Triglycerides (mmol/l)	1.39 (1.23-1.56)	1.11 (1.09-1.25)	0.012
HDL-C (mmol/l)	1.11 (1.04-1.19)	1.20 (1.12-1.27)	0.116
LDL-C (mmol/l)	3.02 (2.76-3.28)	2.70 (2.50-2.90)	0.759
sdLDL-C (mmol/l)	1.38 (1.23-1.53)	0.76 (0.65-0.86)	< 0.001
lbLDL-C (mmol/l)	1.64 (1.41-1.89)	1.94 (1.72-2.16)	0.069
%LDL as sdLDL	47.50 (42.84-52.16)	31.46 (24.97-37.96)	0.001

Values are expressed as mean (95% confidence interval), Unpaired student’s t-test, *P* < 0.05 = significant

Table 6 represents a comparison between the different lipid parameters measured in women who have diabetes and those without diabetes. Except for HDL-C and lbLDL-C, the rest of the lipids and lipoproteins measured should statistically significant difference between the two groups with all the parameters including TC, TG, LDL-C, sdLDL-C and percentage LDL as sdLDL observed to be higher among diabetes subjects. Women with diabetes are more likely to have as much as 44.83% of their LDL-C existing as sdLDL-C.

**Table 6:** Serum lipid concentrations in women with diabetes compared with women without diabetes

Variable	Diabetes	Controls	*P*-value
	(*n* = 116)	(*n* = 85)	
Total cholesterol (mmol/l)	5.37 (5.13–5.61)	4.32 (4.11–4.53)	< 0.001
Triglycerides (mmol/l)	1.43 (1.32–1.55)	0.92 (0.81–1.02)	< 0.001
HDL–C (mmol/l)	1.26 (1.19–1.33)	1.32 (1.22–1.42)	0.317
LDL–C (mmol/l)	3.46 (3.23–3.69)	2.60 (2.40–2.80)	< 0.001
sdLDL–C (mmol/l)	1.48 (1.36–1.60)	0.83 (0.70–0.96)	< 0.001
lbLDL–C (mmol/l)	1.98 (1.77–2.19)	1.77 (1.57–1.96)	0.136
%LDL as sdLDL	44.83 (41.39–48.27)	33.89 (28.86–38.93)	< 0.001

Values are expressed as mean (95% confidence interval), Unpaired student’s t-test, *P* < 0.05 = significant

The diabetic group was divided into 3 subgroups based on their age—Table 7. Subgroup 1 was 35 to 45 years old and amounted to 26(14.8%) in number. Subgroup 2 included those who were 46–60 years old. They were 98 in number and made up 55.7% of the total diabetics. The third subgroup was >60 years, 52 in number and accounted for 29.5% of the total diabetes patients. There was no statistically significant difference in any of the lipid parameters considered across the 3 age groups.

**Table 7:** Comparison of mean lipid parameters among diabetes patients according to age groups

	Subgroup 1	Subgroup 2	Subgroup 3		
Variable/Age (years)	< 46 (*n* = 26)	46-60 (*n* = 98)	> 60 (*n* = 52)	F-value	*p*-value
TC (mmol/L)	4.7 ± 0.9	5.36 ± 1.4	5.01 ± 1.3	3.058	0.050
TG (mmol/L)	1.29 ± 0.6	1.45 ± 0.7	1.43 ± 0.6	0.697	0.499
HDL-C (mmol/L)	1.19 ± 0.2	1.23 ± 0.4	1.18 ± 0.3	0.358	0.700
LDL-C (mmol/L)	2.98 ± 0.9	3.45 ± 1.3	3.21 ± 1.1	1.862	0.158
sdLDL-C (mmol/L)	1.25 ± 0.4	1.46 ± 0.6	1.50 ± 0.7	1.432	0.242
lLDL-C (mmol/L)	1.72 ± 0.6	1.99 ± 0.6	1.69 ± 0.5	1.544	0.216
%LDL-C as dLDL-C	45.70 ± 15.4	44.02 ± 15.5	48.78 ± 16.9	0.896	0.410

Values are expressed as Mean ± SD, ANOVA, *p* < 0.05 = significant

## Discussion

Our data has shown that dyslipidaemia is a problem among type 2 diabetes patients as has been well reported in other similar studies [13–15]. Although there was increase in almost all lipid parameters measured in this study among diabetes patients than controls, the mean TC, TG, LDL-C, and HDL-C in diabetes patients did not meet definitions for dyslipidaemia using criteria such as the National Cholesterol Education Program/Adult Treatment Panel III (NCEP ATP III)] [16]. This observation is in agreement with some previous studies in Nigeria that showed that black Africans have a low prevalence of dyslipidaemia [17]. The high fibre, unrefined carbohydrate diet with low saturated fat commoner among Nigerians may account for this picture. Some researchers have found that diets high in carbohydrates, glycaemic index or both have an adverse relationship to total cholesterol, LDL-C and HDL-C levels with a moderate increase in the overall TC:HDL-C ratio [18]. The implication is that the apparent beneficial effect of a low cholesterol level may not persist in the long run. However, this observation was made in a highly motivated population and the composition of carbohydrates in their diet may be more refined carbohydrates in comparison to the average Nigerian diet. Notably, the TC:HDL-C ratio in our study was found to be lower than what was reported in other studies that involved Whites with no risk factors for CHD [18, 19].

Suppressed HDL-C has been reported as a component of diabetic dyslipidaemia [20, 21]  and an important indicator for an elevated risk of CAD in diabetes even if TC and TG were strictly normal [22]. This study did not however demonstrate a reduction of HDL-C in the diabetes patients. Some researchers have suggested that protective HDL-C was significantly higher in tropical Africa including diabetes patients in Nigeria [14, 23]. The apparently high HDL-C among diabetes patients in this study and others is in keeping with the relatively low-incidence of acute myocardial infarction (AMI) in our environment [23–25]. No participant in this study reported a past episode of AMI. Epidemiological and other studies have consistently confirmed the inverse relationship between HDL-C and CAD [3, 26, 27]. The beneficial effects of HDL are primarily link to their role in reverse cholesterol transport mechanism, i.e., the cholesterol in HDL is being transported back to the liver for excretion out of the body. HDL is also believed to be involved in beneficial mechanisms such as inhibition of lipid peroxidation, cellular adhesion and/or platelet activation resulting in low levels of dyslipidaemia.

This is probably the first study that has estimated the level of LDL sub-fractions in diabetics and non-diabetic controls in our setting. The mean sdLDL-C concentration among the controls in this study compares closely with the findings in a healthy adult population in Japan, where the cut-off for increased sdLDL-C was determined to be >0.9 mmol/l and supported by results from the FOS [28]. Although it may not be wise to apply this cut-off to our subject, the mean value of sdLDL-C among our control subjects was determined to be 0.8 ± 0.54 mmol/L. This may not represent the true value in our population due to the apparently small sample size in this study. Future studies involving larger sample sizes are needed to determine this cut off in our populations. Since it has been established that dyslipidaemia, especially involving increase in sdLDL-C is the basis of atherosclerosis and intima-media thickening of blood vessels [29, 30], we suggest that the dyslipidaemia demonstrated in our study subjects probably contributed to the establishment or maintenance of hypertension in the 75% of diabetes subjects found to be hypertensive. This is much higher than an earlier reported incidence of hypertension among diabetes patients who are Nigerians [31] and indicates that the problem may be increasing. It can also be expected that other unmeasured indices of atherogenesis are likely to worsen in these subjects as long as the blood levels of sdLDL-C and TG remain elevated. Small, dense LDL has been shown to be a marker for atherosclerosis owing to its susceptibility to oxidation and ability to binds more readily to arterial wall proteoglycans. Moreover, it has been shown to be an independent predictor of coronary artery disease in healthy men [19].

Patients with AMI have been observed to show a reduction of LDL size quite early in the course of the event. This abnormality appears to precede all other plasma lipoprotein modifications and is persistent throughout the period of admission [32]. In this study, the level of sdLDL was significantly higher among diabetes subjects than controls. This may have been partly responsible for the few (0.05%) reported cases of transient ischemic attack and stroke among our diabetes subjects. There was no reported case of AMI among our study participants. One reason for the low CVD episodes may be the presence of high HDL-C observed among participants of this study, as explained earlier. The second possible explanation is the diet composition and frequent engagement of our study subjects in some moderate physical activities. However, our findings were in harmony with other researchers who reported a very low incidence of AMI (0.2% of annual adult admissions and 0.04% of all adult admissions) over a 10 year period in Benin, Southern Nigeria [33]. A prevalence of 0.9% for ishaemic heart disease (IHD) and 0.004% for AMI of all medical admissions under a 5 year period was reported in Kano, Northern Nigeria [25]. However, a gradual increase in the number of people presenting with AMI in the University of Benin Teaching Hospital (UBTH) during the study period was also demonstrated [33]. With the recent increase in reported cases of sudden unexplained deaths among Nigerians, there is reason to believe that the present prevalence of CVD will be much higher today. Moreover, the prevalence of sdLDL among healthy non-diabetics in a small Mediterranean Island was determined to be 30 – 35% of total LDL-C in adult men. [34] This study reported 32% of LDL-C as sdLDL-C among non-diabetic men which is almost the same as the Mediterranean study. Among diabetes patients, a slightly higher prevalence of 50% was reported [34] which is higher than the 45% noted in this study.

Improving glycaemic control has been shown to have a favourable effect on lipid and lipoprotein concentrations in type 2 diabetes [35, 36]. However, other researchers have demonstrated that DM subjects with good glycaemic control continued to exhibit a predominance of small, dense LDL particles [37]. This may be due to the presence of hyperinsulinaemia either from insulin resistance or the use of oral hypoglycaemic agents [38]. In this study, diabetes patients were regular clinic attenders and were in good glycaemic control—one criterion considered during patient selection. Since the study was carried out using a one-off measurement of the lipid profile rather than serial measurements, it was not considered necessary to confirm long-term glycaemic control with HbA1c. However, HbA1c measurements would have strengthened our clinical judgment. This is a limitation of this study. Nevertheless, this study has demonstrated that dyslipidaemia is an issue among diabetes patients in our environment. We suggest that early initiation of lipid lowering medications may go a long way to delay or even prevent the development of hypertension and other effects of dyslipidaemia common among type 2 diabetes patients.

## Conclusion

The classical atherogenic lipoprotein phenotype was not demonstrated among subjects with type 2 diabetes mellitus in this study. This may explain to some extent the low incidence of CVD among our diabetes patients. However, dyslipidaemia characterised by an elevation of small, dense LDL-C and triglyceride is a big issue among diabetes patient in our environment and is increasing. If the elevation of small dense low density lipoprotein cholesterol is truly the antecedent of the full blown atherogenic lipoprotein phenotype, it is reasonable to suggest that lipid-lowering medication should be helpful in the management of these patients. A clinical trial of early use of lipid-lowering medication on the basis of elevation of small dense low density lipoprotein cholesterol in diabetes subjects would probably be necessary to support our speculation.

## Abbreviations

ALP: Atherogenic lipoprotein phenotype
AMI: Acute myocardial infarction
ANOVA: Analysis of variance
APIN: AIDS Prevention in Nigeria
Apo B: Apolipoprotein B
BMI: Body Mass Index
CAD: Coronary artery disease
CHD: Coronary heart disease
CI: Confidence interval
CVD: Cardiovascular disease
DM: Diabetes mellitus
FOS: Framingham offspring study
HDL-C: High density lipoprotein cholesterol
IHD: Ischemic heart disease
JUTH: Jos University Teaching Hospital
lbLDL-C: Large buoyant low density lipoprotein cholesterol
LDL-C: Low density lipoprotein cholesterol
MOPD: Medical out-patient department
NCEP ATP III: National Cholesterol Education Program- Adult Treatment Panel III
SD: Standard deviation
sdLDL-C: small, dense low density lipoprotein cholesterol
TC: Total cholesterol
TG: Triglyceride
TIA: Transient ischemic attack
UBTH: University of Benin Teaching Hospital
VLDL-C: Very low density lipoprotein cholesterol
